# Mode-dependent differences in kinetic and selected temporospatial gait parameters in healthy cats walking on a pressure-sensitive treadmill: a pilot study

**DOI:** 10.3389/fvets.2026.1816089

**Published:** 2026-08-19

**Authors:** Monika Anna Mille, Dorothea Weissig, Britta Dobenecker, Erik Björn Mille, Yury Zablotski, Susanne Katja Lauer

**Affiliations:** 1 LMU Small Animal Clinic, Centre for Clinical Veterinary Medicine, LMU Munich, Munich, Germany; 2 Diagnostic Centre for Small Animals, Dresden, Germany; 3 Chair of Animal Nutrition and Dietetics, Department of Veterinary Sciences, LMU Munich, Munich, Germany; 4 Federal Office for Radiation Protection, Oberschleißheim, Germany

**Keywords:** feline gait analysis, feline locomotion, kinetics, pressure-sensitive treadmill, temporospatial parameters, treadmill

## Abstract

**Background:**

Standardized and repeatable assessment of feline gait remains challenging, highlighting the need for controlled measurement systems such as pressure-sensitive treadmills.

**Objectives:**

This study aimed to compare kinetic and temporospatial gait parameters in healthy cats walking on a treadmill operated in standing vs. running mode. We hypothesized that no significant differences would be observed between modes.

**Methods:**

Ten healthy adult cats were enrolled in a prospective, controlled crossover study. Inclusion criteria included body weight >4.0 kg, body condition score (BCS) 4–6/9, and age >24 months. Cats walked on a pressure-sensitive treadmill (FDM-T-CanidGait^®^, zebris Medical GmbH, Isny, Germany) under two conditions: standing mode (stationary belt) and running mode (moving belt). Five 2-min trials per mode were recorded. Kinetic and temporospatial gait parameters were assessed, including peak vertical force, vertical impulse, stride length, stance and swing phase duration, and step width. Right and left limb data were pooled and analyzed collectively as forelimbs (FL) and hindlimbs (HL). Data were analyzed using robust linear mixed-effects models (R 4.4.3), with significance set at *p* < 0.05.

**Results:**

Significant differences between treadmill modes were detected for peak vertical force and vertical impulse in both forelimbs and hindlimbs (*p* < 0.01). Forelimb swing and stance phase durations also differed significantly (*p* < 0.05). In contrast, stride length, step width, and hindlimb temporal parameters did not differ significantly between modes. Estimated differences were generally small in magnitude across variables.

**Conclusion:**

In healthy cats, stride length, step width, and hindlimb temporal parameters were comparable between standing and running treadmill modes. In contrast, kinetic variables (peak vertical force and vertical impulse) and forelimb stance and swing phase durations differed significantly between modes and should therefore be interpreted only within the same treadmill mode. These findings indicate that treadmill modes may be used interchangeably for selected temporospatial parameters but not for kinetic variables or forelimb temporal parameters.

## 1 Introduction

Subjective gait assessment in cats remains challenging because species-specific behavioral characteristics, sensitivity to environmental changes, and variable cooperation limit standardized locomotor evaluation (1–5). In addition, visual gait assessment often fails to detect subtle locomotor asymmetries or mild lameness, as cats frequently compensate efficiently and may exhibit only minimal overt gait alterations during routine examination (4, 6–8). Consequently, objective gait analysis techniques have gained increasing importance in feline locomotor research.

Previous feline gait analysis studies have predominantly employed pressure-sensitive walkway or platforms to quantify kinetic and temporospatial gait parameters during overground locomotion (6–8). Beyond establishing reference values for peak vertical force (PVF), vertical impulse (VI), and temporospatial gait variables in healthy cats, these systems have also enabled objective assessment of locomotor adaptations during jumping and voluntary gait modification (6–8).

Pressure-sensitive walkway analysis has further been used to characterize gait following surgical interventions in cats. More than 6 months after bilateral forepaw onychectomy, no differences in PVF or VI were detected compared with non-onychectomized cats (9). By comparison, cats evaluated 1 year after femoral head and neck ostectomy exhibited persistently reduced PVF and VI, whereas those treated with total hip replacement showed improved limb loading and gait symmetry (7, 9, 10).

Despite their established role in feline gait research, overground pressure-sensitive walkways have inherent methodological limitations. Variability in walking velocity between trials, the restricted recording area limiting the number of valid footfalls per pass, and low trial efficiency often require repeated measurements to obtain sufficient valid data (11–13). In cats, these limitations are further compounded by inconsistent walking behavior including intermittent stopping or turning, and variable motivation, which complicate data standardization and may increase within-subject variability.

As an alternative to overground gait analysis, treadmill-based gait analysis become an established research tool in small animal locomotor research, with most methodological developments originating from canine studies (14, 15). Treadmill systems provide controlled walking speed, continuous data acquisition, and the collection of multiple consecutive gait cycles within a confined measurement area (16, 17). In addition, instrumented treadmill systems can be integrated with motion capture systems (18) and electromyographic recordings (19), enabling simultaneous assessment of kinetic, kinematic, and neuromuscular aspects of locomotion.

In dogs, direct comparisons between treadmill and overground locomotion have demonstrated parameter-dependent differences, indicating that the two conditions cannot be considered directly interchangeable (13). Compared with overground locomotion, treadmill walking is characterized by longer stance phase duration, shorter swing phase duration, and stride length, whereas PVF and VI show excellent agreement during trotting (13, 16).

In cats, treadmill-based locomotor research has largely been confined to experimental and neurophysiological studies (20–23). Only one early study compared treadmill walking with overground locomotion in cats, identifying differences in temporal gait characteristics but without evaluating kinetic parameters (22). More recently, a pressure-sensitive treadmill was shown to provide repeatable and reliable kinetic and temporospatial gait measurements in healthy cats under standardized conditions (24) and to enable rapid, feasible, and standardized gait assessment with consistent data acquisition across a large cohort of clinically normal cats (25). However, whether treadmill-derived gait parameters are directly comparable to those obtained during overground locomotion remains unknown. This question is of practical importance because variable feline cooperation may necessitate the use of both overground and treadmill-based gait assessment within the same study or during repeated evaluations of the same animal (16).

The aim of the present study was to compare kinetic and temporospatial gait parameters between standing and running treadmill mode in healthy adult cats. Walking on a treadmill without belt motion was used as a surrogate for walkway-based assessment within the same measurement system, thereby avoiding methodological variability associated with cross calibration. We hypothesized that no significant differences would be detected between the two treadmill modes, which would allow flexible switching between standing and running treadmill configurations, when cats exhibit variable compliance across sessions, while maintaining comparability of gait data in clinical and experimental settings.

## 2 Materials and methods

### 2.1 Cats

Healthy adult cats were enrolled in this prospective study, including client-owned animals and cats housed at the Chair of Animal Nutrition and Dietetics, Ludwig-Maximilians-University Munich. Written informed consent was obtained from the owners of all client-owned cats prior to participation. A subset of the data analyzed in the present study was obtained from cats that had previously participated in a separate investigation using the same treadmill system and general experimental framework (24).

Eligibility for inclusion required the absence of orthopedic or neurologic abnormalities, as determined by medical history, standardized orthopedic and neurologic examination, and subjective gait evaluation. Cats were required to have a minimum body weight of 4.0 kg and a body condition score between 4 and 6 on a 9-point scale (26). Cats were excluded if infectious, immune-mediated, or clinically relevant cardiac disease was suspected, or if analgesic or non-steroidal anti-inflammatory medication had been administered within 14 days prior to data acquisition. Only cats that walked voluntarily on the treadmill after habituation and showed no signs of stress or reluctance during data acquisition were included.

The study was conducted in accordance with institutional and national guidelines for animal welfare. The study was approved by the Ethics Committee of the Center for Veterinary Medicine, Ludwig-Maximilians-University Munich (approval number 218-16-06-2020). Data collection was conducted within the approved study period, which was extended and remained valid until February 2025.

### 2.2 Treadmill setup

Gait assessment was performed using a pressure-sensitive treadmill system (FDM-T-CanidGait^®^, zebris Medical GmbH, Isny, Germany). The pressure-sensitive surface measured 190 × 46 cm and comprised 9,216 capacitive sensors with a measurement range of 0.5–120 N/cm^2^. Data were recorded at a sampling frequency of 100 Hz using the manufacturer's proprietary software (Animal Analysis Suite, version RC 2.3.28). Elevated entry and exit platforms (60 × 45 × 24 cm) were positioned at the rear and front of the treadmill to facilitate voluntary and continuous walking. Two synchronized video cameras (SYNCLightCam; WinFDM Software version 1.2.2, zebris Medical GmbH) were used to obtain lateral and caudal views for visual verification of gait consistency and subsequent step selection. Recordings were conducted in two indoor rooms corresponding to the locations of client-owned and institution-housed cats. Environmental conditions, including lighting, ambient noise, and room temperature, were kept as constant as possible within each location to minimize external influences on locomotion.

### 2.3 Gait assessment

Each cat completed two assessment sessions approximately 2 weeks apart. Prior to data acquisition, cats were allowed to acclimate to the environment and treadmill for up to 15 min, with a maximum acclimatization period of 30 min. The total duration per session did not exceed 60 min. Data acquisition consisted of five structured trials of 2 min each and was therefore intermittent, accounting for only a limited proportion of the overall session duration.

#### 2.3.1 Gait assessment session 1

During the first session, gait data were collected in standing treadmill mode with a stationary belt, which was used as a formal measurement condition. For each trial, the cat was positioned on an elevated platform at the rear of the treadmill and encouraged to walk forward toward a handler located at the front. Motivation was provided using toys, food rewards, clickers, or verbal cues, selected according to individual preference. Five valid trials were recorded. Throughout data collection, only trials demonstrating a consistent walking gait were considered valid. Trials showing gait transitions, pacing, trotting, stopping, or turning were excluded. Based on these trials, an individual mean walking speed was calculated for each cat and used to define the target speed for treadmill walking in gait assessment session 2.

Following completion of data collection in standing treadmill mode, cats were habituated to the moving treadmill during the same session. The belt was initially activated at a low speed and subsequently increased in increments of 0.1 km/h until the cat demonstrated relaxed and steady walking behavior. Alternatively, cats were placed directly onto the moving treadmill if this facilitated initiation of locomotion.

#### 2.3.2 Gait assessment session 2

During the second session, gait data were collected in running treadmill mode using the individually determined walking speed established during the first session to avoid speed dependent effects. All procedural aspects were kept consistent between sessions. If gait recordings obtained during the standing treadmill mode in the first session did not meet the predefined quality control criteria (e.g., insufficient number of valid strides, inconsistent gait patterns such as stopping or irregular stepping, crouching, or exaggerated movements of the head, tail, or ears) for at least five consecutive strides, cats were allowed to repeat the stationary treadmill trials prior to data acquisition in running treadmill mode to obtain adequate recordings. No further adjustments to the treadmill speed were made.

Data obtained during the running treadmill mode were also used for separate analyses addressing the repeatability and reliability of kinetic and center of pressure parameters, which have been reported elsewhere (24). Trials were repeated if unexpected disturbances occurred. The total duration per cat, including familiarization and data acquisition, did not exceed 60 min per session.

### 2.4 Data analysis

Kinetic and temporospatial gait parameters were calculated for all four limbs, including PVF, VI, stride length, swing phase duration, stance phase duration, step width and the symmetry indices of PVF and VI of each limb (27). Symmetry index was calculated as


$$\begin{array}{l} \text{SI}\left(\%\right)=\frac{X_{r}-X_{l}}{X_{r}+X_{l}}\times \;200\left(28\right) \end{array}$$

where *X*_*r*_ and *X*_*l*_ represent the values obtained from the right and left limb, respectively. All recordings were processed using the manufacturer's proprietary software (Animal Analysis Suite, Beta version 2.4.4; zebris Medical GmbH, Isny, Germany). For each cat and assessment session, synchronized video recordings were reviewed to identify valid walking sequences. Five valid sequences per session were selected for analysis. In standing treadmill mode, the fixed length of the pressure-sensitive surface resulted in two to four gait cycles per trial, depending on individual stride length and the position of initial paw contact. In running treadmill mode, five trials were selected, each comprising three consecutive gait cycles. Sequences were included only when cats maintained a consistent walking speed and displayed stable forward locomotion. Trial selection criteria were further refined to differentiate between minor movements (e.g., subtle ear or tail motion) and movements affecting gait (e.g., head turning, sudden deceleration, or limb interference). These criteria were applied by an experienced investigator trained in gait analysis (ECVSMR resident/diplomate). Movements of the head, tail, or ears were evaluated during video review; sequences with pronounced movements were excluded, whereas minor ear or tail movements were considered acceptable.

### 2.5 Statistical analysis

All statistical analyses were performed using R [version 4.4.3; R Foundation for Statistical Computing; (48)]. Model fitting and inference were conducted using the packages *lme4* (28), *robustlmm* (29), *emmeans* (30), and *performance* (31). Prior to model selection, the distribution of each outcome variable was evaluated by visual inspection of quantile–quantile plots and by application of the Shapiro–Wilk test. Linear mixed-effects models and robust linear mixed-effects models were fitted for each gait parameter and compared with respect to model assumptions and overall performance. As several parameters deviated from normality, and robust models demonstrated superior performance, robust linear mixed-effects models were retained for all final analyses. For subsequent analyses, data from right and left limbs were combined based on the assumption of approximate symmetry of gait parameters in healthy cats (6, 32). To support this assumption, a laterality analysis was performed using a robust linear mixed-effects model with laterality specified as a fixed effect and cat identity as a random effect. For each treadmill mode, left-right differences were evaluated using estimated marginal means, with forelimbs and hindlimbs analyzed separately. In the pooled analysis, treadmill mode was included as a fixed effect and cat identity as a random effect to account for repeated measurements within individuals.

Pairwise comparisons between treadmill modes were performed using estimated marginal means. Forelimb and hindlimb data were analyzed separately. Adjustment for multiple comparisons was applied using the Tukey method, and statistical significance was defined as a *p*-value < 0.05. Model results are reported as estimated marginal means with corresponding 95% confidence intervals.

## 3 Results

### 3.1 Study population and data quality

Ten of fifteen cats successfully completed both the standing and running treadmill conditions with evaluable gait sequences and were therefore included in the final analysis (Supplementary Table 1). Several cats did not meet all predefined quality control criteria during the standing treadmill condition. Common reasons for exclusion included an insufficient number of valid steps, stopping, or jumping off the treadmill. Three cats required repeated trials during the second assessment of the standing treadmill condition, as one, two, and three trials, respectively, were excluded due to quality control criteria (i.e., irregular gait patterns or head movements).

The final cohort comprised six client-owned cats and four cats housed at the Chair of Animal Nutrition and Dietetics, Ludwig-Maximilians-University Munich. Cats had a mean body weight of 4.6 ± 0.4 kg and a mean age of 5.6 ± 1.3 years at the time of inclusion. Breeds included European Shorthair (*n* = 8), European Longhair (*n* = 1), and British Shorthair (*n* = 1). Nine cats were male (three intact and six neutered), and one cat was a spayed female. The mean walking speed determined during the standing treadmill condition was 2.32 ± 0.32 km/h and was used unchanged for the running treadmill condition for each cat. Detailed individual data are provided in the Supplementary material.

The laterality analysis (Supplementary Table 2) revealed no relevant asymmetry for any parameter except hindlimb stride length. This parameter showed a statistically significant left–right difference, the magnitude of this effect was minimal (approximately 3%) and was considered not clinically relevant, supporting the combined analysis of left and right limbs. In addition, symmetry indices for PVF and VI showed no significant differences, further supporting the validity of pooling left and right limb data (Supplementary Table 3). These data are also provided in Supplementary material.

### 3.2 Kinetic and temporospatial parameters

**Figure 1:**
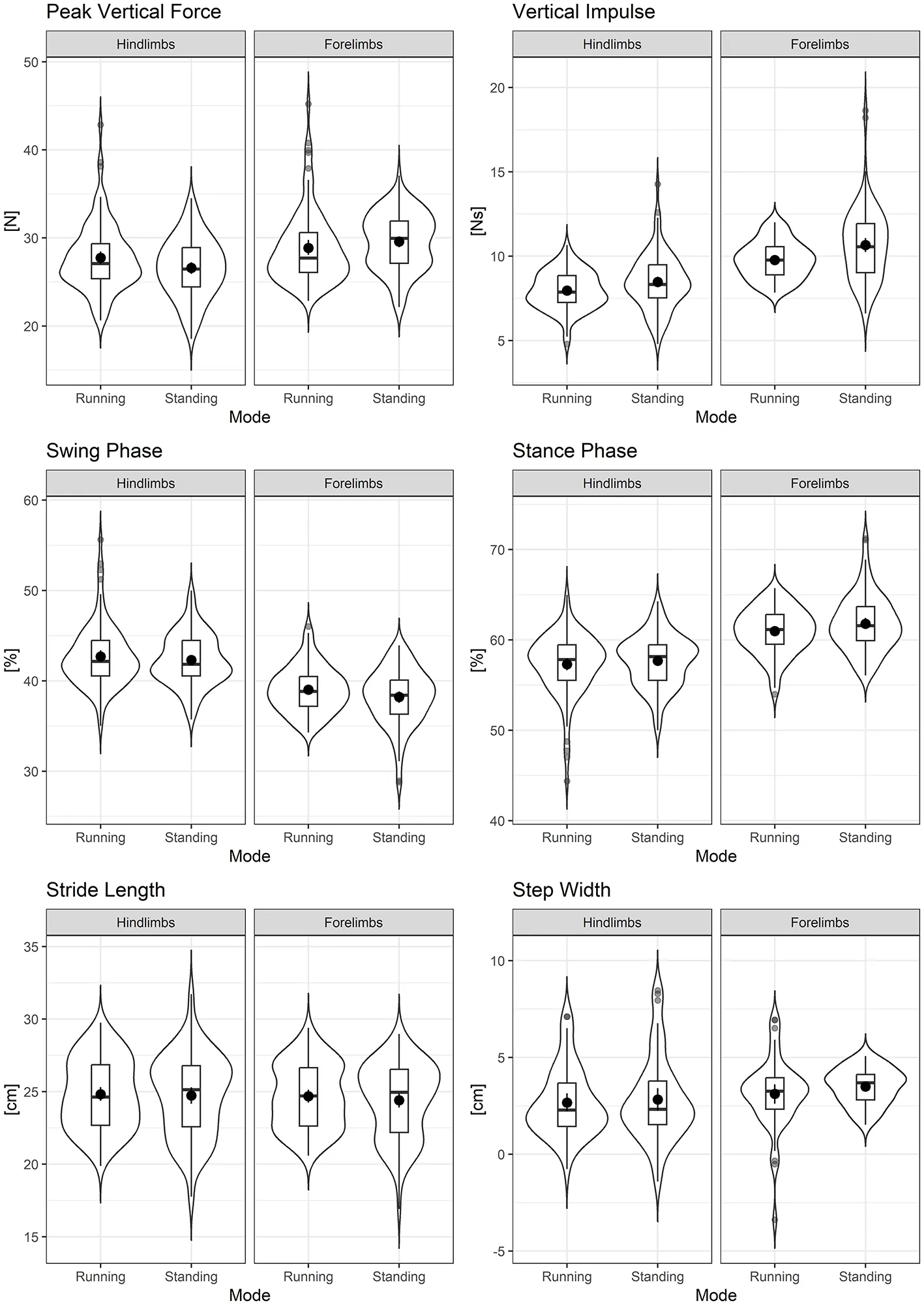
Comparison of kinetic and temporospatial gait parameters between standing and running treadmill mode for forelimbs and hindlimbs. Boxplots depict medians and interquartile ranges, violin plots illustrate the data distribution and dots with whiskers represent means with bootstrapped 95% confidence levels (*n* = 10).

A descriptive comparison of gait parameters between treadmill modes is given in Figure 1.

**Figure 2:**
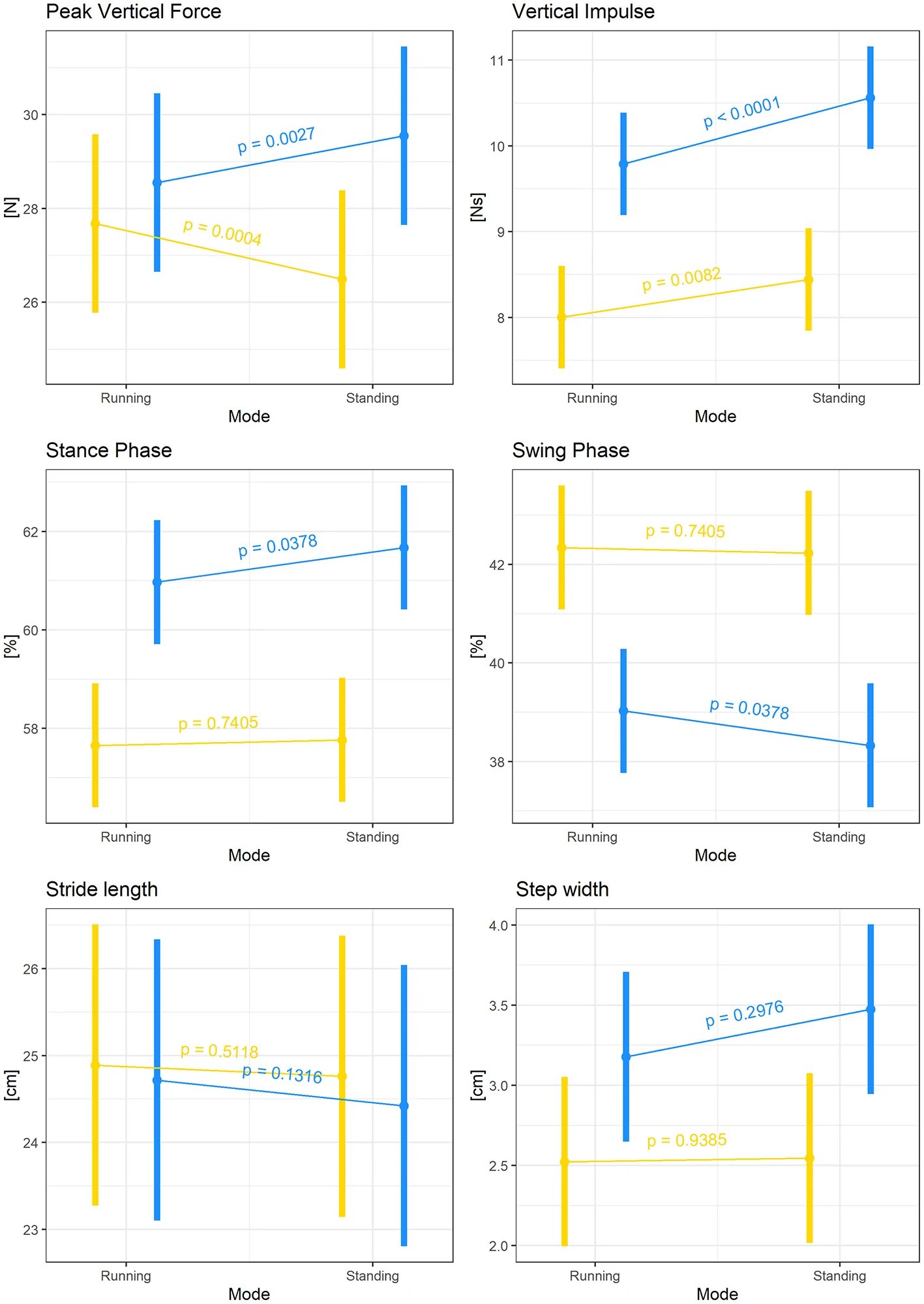
Comparison of kinetic and temporospatial gait parameters between standing and running treadmill modes for forelimbs (blue) and hindlimbs (yellow), based on predicted estimated marginal means from robust linear mixed-effects models with corresponding 95% confidence intervals (*n* = 10).

Estimated marginal means with 95% confidence intervals, effect sizes as estimated mean differences (Δ) with 95 % confidence intervals, and *p*-values for all gait parameters are presented in Table 1 and visualized in Figure 2. Differences between treadmill modes were observed primarily for kinetic parameters, whereas less temporospatial parameters showed differences (Figures 1, 2).

**Table 1:** Comparison of kinetic and temporospatial gait parameters between standing and running treadmill modes in healthy cats (*n* = 10).

Parameter	Limb	Treadmill mode				Effect size		*p*-Value
		Standing		Running				
		Mean	95% CI	Mean	95%CI	Δ	95% CI	
Peak vertical force [N]	FL	29.6	27.7–31.4	28.6	26.7–30.4	−0.999	−1.650 to −0.347	0.0027
	HL	26.5	24.6–28.4	27.7	25.8–29.6	1.186	0.534–1.837	0.0004
Vertical impulse [Ns]	FL	10.56	9.97–11.16	9.79	9.19–10.39	−0.772	−1.099 to −0.445	< 0.0001
	HL	8.44	7.85–9.04	8.00	7.41–8.60	−0.441	−0.768 to 0.114	0.0082
Stance phase duration [% gait cycle]	FL	61.7	60.4–62.9	61.0	59.7–62.2	−0.704	−1.369 to −0.0398	0.0378
	HL	57.8	56.5–59.0	57.7	56.4–58.9	−0.112	−0.777 to 0.5521	0.7405
Swing phase duration [% gait cycle]	FL	38.3	37.1–39.6	39.0	37.8–40.3	0.704	0.0398–1.369	0.0378
	HL	42.2	41.0–43.5	42.3	41.1–43.6	0.112	−0.5521 to 0.777	0.7405
Stride length [% gait cycle]	FL	24.4	22.8–26.0	24.7	23.1–26.3	0.295	−0.0884 to 0.678	0.1316
	HL	24.8	23.1–26.4	24.9	23.3–26.5	0.128	−0.2549 to 0.511	0.5118
Step width [cm]	FL	3.47	2.95–4.00	3.18	2.65–3.70	−0.297	−0.855 to 0.262	0.2976
	HL	2.55	2.02–3.07	2.52	2.00–3.05	−0.022	−0.580 to 0.536	0.9385

Robust linear mixed-effects models were used. Results are presented as estimated marginal means (Mean) with 95% confidence intervals (CI), estimated mean differences (Running–Standing) with 95% CI and p-values. FL, forelimb; HL, hindlimb.

#### 3.2.1 Peak vertical force

In the forelimbs, PVF was significantly lower during the running treadmill mode compared with standing mode (*p* = 0.0027), corresponding to a mean estimated difference of 3.3% relative to baseline values. In the hindlimbs, PVF was significantly higher during the running treadmill mode (*p* = 0.0004), corresponding to a mean estimated difference of 4.4% relative to baseline. Both effects were small in magnitude.

#### 3.2.2 Vertical impulse

In the forelimbs, VI was significantly lower during the running treadmill mode compared with standing mode (*p* < 0.0001), corresponding to a mean estimated difference of 7.3% relative to baseline values. In the hindlimbs, VI was significantly lower during the running treadmill mode (*p* = 0.0082), with a mean estimated reduction of 5.2% relative to baseline. Effects were small to moderate in magnitude.

#### 3.2.3 Stance phase and swing phase duration

In the forelimbs, stance phase duration was significantly shorter during the running treadmill mode compared with standing mode (*p* = 0.0378), corresponding to a mean estimated difference of 1.1% relative to baseline values (Figure 2C). Conversely, swing phase duration in the forelimbs was significantly longer during the running treadmill mode (*p* = 0.0378), with a mean estimated difference of 1.8%. Both effects were associated with small magnitudes.

In the hindlimbs, neither stance nor swing phase duration differed significantly between treadmill modes (*p* = 0.7405 for both parameters), with mean estimated differences below 0.5% corresponding to small effects.

#### 3.2.4 Stride length and step width

Stride length did not differ significantly between treadmill modes in either the forelimbs (*p* = 0.13) or the hindlimbs (*p* = 0.51), with mean estimated differences corresponding to 1.2 and 0.5% of baseline values, respectively and small effects.

Step width did not differ significantly between treadmill modes in either the forelimbs (*p* = 0.30) or the hindlimbs (*p* = 0.94), with mean estimated differences corresponding to 8.6 and 0.9% of baseline values, respectively.

## 4 Discussion

### 4.1 Principal findings

This study represents the first systematic comparison of kinetic and temporospatial gait parameters in healthy cats walking on a pressure-sensitive treadmill operated in standing and running treadmill modes. Although most of the evaluated temporospatial parameters remained comparable between treadmill modes, statistically significant mode-dependent differences were identified for PVF and VI, with mean differences ranging from approximately 3.3%−7.3%, as well as for forelimb stance and swing phase durations. In the context of objective gait analysis, differences in this range are commonly considered borderline to clinically relevant, as deviations exceeding approximately 5% are often associated with clinically detectable asymmetry (33). In contrast, stride length, step width, and hindlimb temporal parameters were largely unaffected by treadmill motion.

Overall, these findings indicate that while most feline gait parameters remained similar between modes, treadmill mode exerted a measurable influence on specific aspects of gait.

### 4.2 Clinical implications

Recent studies in dogs have confirmed that treadmill and overground locomotion differ in multiple temporospatial, kinematic and kinetic parameters, reinforcing the need for condition-specific interpretation (16, 34). From a clinical perspective, these findings are particularly relevant when subtle gait alterations are of interest. Mode-dependent differences in kinetic parameters and forelimb timing may influence the detection of mild lameness or the interpretation of longitudinal changes during follow-up examinations. Consequently, consistent use of treadmill mode appears essential when gait parameters are used for clinical decision-making or monitoring of disease progression.

### 4.3 Interpretation of mode-dependent differences

The increased PVF and VI observed in the hindlimbs during running treadmill mode may reflect altered propulsive demands associated with belt-driven locomotion (35). Although direct comparisons between treadmill and overground walking in cats are limited, studies in dogs demonstrate that hindlimbs play a dominant role during treadmill walking with increased PVF, VI and hindlimb loading reflecting their contribution to forward progression on a moving belt (13, 16, 36–38). By analogy, the increased hindlimb loading observed in cats during running treadmill mode may represent subtle biomechanical adaptations to treadmill walking. Conversely, the reduction in forelimb PVF and VI during running treadmill mode suggests a relative redistribution of load toward the hindlimbs. Given that PVF and VI are commonly used as key outcome measures in feline lameness assessment (33), these differences may be clinically relevant and should be interpreted with caution, particularly when comparing data obtained under different measurement conditions. In light of the well-documented adaptability of feline locomotion and postural control, modest changes in environmental constraints may lead to measurable kinetic alterations without overt changes detectable by visible gait assessment (39, 40). From a rehabilitation perspective, these mode-specific characteristics may be of practical relevance, as different treadmill settings could be used in a targeted manner. For example, running treadmill mode may promote hindlimb engagement and propulsion, whereas stationary treadmill walking may more closely resemble overground conditions and could be preferable for initial gait assessment or for patients with limited tolerance to dynamic perturbations. In human medicine, treadmill-based training has been shown to be safe and effective for improving balance and reducing fall risk in elderly populations, including patients with Parkinson's disease, spinal cord injuries, and age-related balance impairments (41). Given these established benefits in human rehabilitation, similar principles may potentially be relevant for feline patients with orthopedic or neurological disorders, where controlled treadmill-based exercise could support functional recovery and mobility training.

Forelimb temporal parameters were more sensitive to treadmill mode than hindlimb timing, as evidenced by significant differences in forelimb stance and swing phase durations, while hindlimb temporal parameters remained unchanged. This limb-specific response is consistent with the distinct functional roles of the limbs during quadrupedal locomotion, with forelimbs primarily contributing to braking and weight support and hindlimbs to propulsion (42, 43). Locomotion on a moving belt may therefore preferentially influence forelimb timing, while hindlimb temporal patterns remain stable. As with the kinetic findings, the magnitude of these differences was small, suggesting limited clinical relevance in routine gait assessment but potential relevance in research settings focusing on subtle temporal changes.

The observed kinetic differences highlight the importance of treadmill mode consistency when absolute force-related parameters are of interest. When PVF or VI are used as primary outcome measures, combining data from standing and running treadmill modes may introduce systematic bias. These findings suggest that treadmill mode should be considered explicitly when interpreting kinetic outcomes, particularly in studies aiming to detect subtle changes associated with disease or therapeutic effects.

The use of a standing treadmill mode within the same measurement system allowed comparison of gait parameters across treadmill configurations without confounding effects related to cross-platform calibration or repeated habituation to different testing environments. The finding that most of the evaluated temporospatial parameters were comparable between standing and running treadmill modes indicates that the influence of treadmill mode is parameter-dependent. The two configurations cannot be considered fully interchangeable, especially in situations where precise gait assessment is required. One limitation of this approach is that direct comparison with true overground walking remains necessary to determine whether treadmill-derived parameters accurately reflect natural gait, especially in cats with orthopedic or neurologic disease, where compensatory strategies and gait asymmetries may be more pronounced.

### 4.4 Study limitations

The relatively small sample size of 10 cats reflects the exploratory nature of the investigation and is consistent with cohort sizes commonly used in pilot gait studies (44, 45). The study population was predominantly male, as fewer female cats met the minimum body weight required to ensure adequate sensor sensitivity of the pressure-sensitive treadmill. Although this imbalance may introduce a degree of selection bias, previous studies using pressure-sensitive walkways have not identified clinically relevant sex-related differences in feline gait parameters, with longer stride length reflecting body size rather than sex (32).

The use of two measurement sessions may have introduced day-dependent variability. This approach was necessary to determine each cat's individual average walking velocity, which was subsequently used to standardize treadmill speed and reduce within-subject variability. A stationary treadmill configuration with entrance and exit platforms was employed to approximate the function of a pressure-sensitive walkway. As a further limitation, acceleration data were not recorded, and transient speed-related effects during gait initiation and termination cannot be fully excluded.

Learning effects and procedural adaptation may have occurred both within and between sessions. In dogs, repeated treadmill exposure has been shown to induce habituation effects within minutes, particularly affecting stance phase duration and PVF, and comparable adaptive responses may also be present in cats (46, 47). To facilitate continuous walking, motivational aids were used and occasionally elicited head or limb movements. As an additional limitation, minor influences of motivational aids on gait patterns cannot be entirely excluded, despite review of all recordings using synchronized video footage and inclusion of only regular and consistent gait cycles without visible disturbances. In a small subset of cats, additional trials were required within the same session to obtain sufficient valid recordings. Although rest periods were provided between trials and fatigue was considered unlikely, a subtle influence of fatigue on gait parameters cannot be entirely excluded and should be acknowledged as a potential limitation.

## 5 Conclusion

In conclusion, clinically relevant differences were observed in PVF and VI, as well as in forelimb stance and swing phase durations, indicating that treadmill mode has a measurable influence on key gait variables. Both treadmill modes can be used for feline gait analysis, but they are not directly interchangeable, particularly when kinetic parameters are used for clinical assessment or detection of subtle lameness. Consistent use of treadmill mode is therefore recommended to ensure reliable interpretation of gait data, especially in longitudinal or clinical settings.

## Data Availability

The original contributions presented in the study are included in the article/Supplementary material, further inquiries can be directed to the corresponding author.
