# Supplementary material for: Mode-dependent differences in kinetic and selected temporospatial gait parameters in healthy cats walking on a pressure-sensitive treadmill: a pilot study
Source: Front Vet Sci. 2026 Aug 19;13:1816089. doi: 10.3389/fvets.2026.1816089 (PMC13536722; doi:10.3389/fvets.2026.1816089)
Supplement: Supplementary file 1 [file Table_1.docx]

| Cat | Sex / Neuter status | Breed | Background | Body weight (kg) | Age (years) | Walking speed (km/h) |
| --- | --- | --- | --- | --- | --- | --- |
| 1 | Male, neutered | ESH | Research colony | 4.62 | 3.5 | 2.60 |
| 2 | Male, neutered | ESH | Research colony | 4.01 | 3.8 | 2.50 |
| 3 | Male, intact | ESH | Research colony | 5.05 | 5.1 | 2.60 |
| 4 | Male, intact | ELH | Client-owned | 4.86 | 6.7 | 2.30 |
| 5 | Male, neutered | ESH | Research colony | 4.38 | 6.5 | 1.80 |
| 6 | Male, intact | ESH | Client-owned | 4.10 | 1.9 | 2.40 |
| 7 | Male, intact | ESH | Research colony | 4.80 | 4.5 | 1.80 |
| 8 | Male, neutered | ESH | Research colony | 4.71 | 5.9 | 2.30 |
| 9 | Female, spayed | ESH | Client-owned | 4.20 | 6.0 | 2.10 |
| 10 | Male, neutered | BSH | Client-owned | 5.28 | 4.1 | 2.80 |

**Supplementary Table 1.** Individual characteristics of all included cats (n = 10), including signalment, body weight, age, and individually determined walking speed. **Legend:**
ESH = European Shorthair; ELH = European Longhair; BSH = British Shorthair.
*Background:* “Research colony” refers to cats housed at the Chair of Animal Nutrition and Dietetics, Ludwig-Maximilians-University Munich. “Client-owned” refers to privately owned cats presented by their owners.

**Supplementary Table 2.** Comparison of laterality between right and left fore- and hindlimbs in standing and running treadmill modes in healthy cats (n = 10). Robust linear mixed-effects models were used to evaluate the effect of limb side (right vs. left) on the investigated parameters. Results are presented as estimated marginal means (Mean) with 95% confidence intervals (CI), estimated mean differences (left-right), and p-values. FL: Forelimb; HL: Hindlimb.

| Parameter | Limb | Treadmill Mode | Laterality | | | | Effect Size  Δ | p- value |
| --- | --- | --- | --- | --- | --- | --- | --- | --- |
|  |  |  | Right | | Left | |  |  |
|  |  |  | Mean | 95% CI | Mean | 95% CI |  |  |
| Peak vertical force  [N] | FL | Standing | 29.4 | 27.4 – 31.3 | 29.7 | 27.8 – 31.7 | 0.370 | 0.4301 |
|  |  | Running | 28.8 | 26.8 – 30.7 | 28.3 | 26.4 – 30.3 | -0.456 | 0.3302 |
|  | HL | Standing | 26.4 | 24.5 – 28.4 | 26.5 | 24.6 – 28.5 | 0.104 | 0.8245 |
|  |  | Running | 27.6 | 25.6 – 29.5 | 27.8 | 25.8 – 29.7 | 0.177 | 0.7063 |
| Vertical impulse  [Ns] | FL | Standing | 10.55 | 9.91 – 11.19 | 10.58 | 9.94 – 11.22 | 0.0307 | 0.8964 |
|  |  | Running | 9.87 | 9.23 – 10.51 | 9.70 | 9.07 – 10.34 | -0.1653 | 0.4831 |
|  | HL | Standing | 8.37 | 7.73 – 9.01 | 8.52 | 7.88 – 9.15 | 0.1415 | 0.5483 |
|  |  | Running | 7.90 | 7.26 – 8.54 | 8.09 | 7.45 – 8.73 | 0.1931 | 0.4128 |
| Stance phase duration  [% gait cycle] | FL | Standing | 61.8 | 60.5 – 63.2 | 61.5 | 60.2 – 62.9 | -0.312 | 0.5161 |
|  |  | Running | 61.4 | 60.0 – 62.7 | 60.6 | 59.2 – 61.9 | -0.790 | 0.1000 |
|  | HL | Standing | 57.6 | 56.2 – 58.9 | 58.0 | 56.6 – 59.3 | 0.404 | 0.4005 |
|  |  | Running | 57.5 | 56.2 – 58.9 | 57.8 | 56.4 – 59.1 | 0.209 | 0.6634 |
| Swing phase duration  [% gait cycle] | FL | Standing | 38.2 | 36.8 – 39.5 | 38.5 | 37.1 – 39.8 | 0.312 | 0.5161 |
|  |  | Running | 38.6 | 37.3 – 40.0 | 39.4 | 38.1 – 40.8 | 0.790 | 0.1000 |
|  | HL | Standing | 42.4 | 41.1 – 43.8 | 42.0 | 40.7 – 43.4 | -0.404 | 0.4005 |
|  |  | Running | 42.5 | 41.1 – 43.8 | 42.2 | 40.9 – 43.6 | -0.209 | 0.6634 |
| Stride length  [% gait cycle] | FL | Standing | 24.4 | 22.7 – 26.0 | 24.5 | 22.9 – 26.1 | 0.1164 | 0.6691 |
|  |  | Running | 24.5 | 22.8 – 26.1 | 24.9 | 23.3 – 26.6 | 0.4621 | 0.0897 |
|  | HL | Standing | 24.7 | 23.1 – 26.4 | 24.8 | 23.2 – 26.4 | 0.0457 | 0.8667 |
|  |  | Running | 24.5 | 22.8 – 26.1 | 25.3 | 23.7 – 27.0 | 0.8740 | 0.0013 |

| Parameter | Limb | Treadmill Mode | | | | Effect Size | | p- value |
| --- | --- | --- | --- | --- | --- | --- | --- | --- |
|  |  | Standing | | Running | |  |  |  |
|  |  | Mean | 95% CI | Mean | 95% CI | Δ | 95% CI |  |
| Symmetry Indices PVF [%] | FL | -0.757 | -3.10 – 1.58 | 1.212 | -1.13 – 3.55 | 1.97 | -0.498 – 4.436 | 0.1178 |
|  | HL | 0.525 | -1.82 – 2.87 | -1.147 | -3.49 – 1.19 | -1.67 | -4.140 – 0.795 | 0.18410 |
| Symmetry Indices VI [%] | FL | 0.658 | -2.62– 3.936 | 1.617 | -1.66 – 4.895 | 0.959 | -2.49 – 4.41 | 0.5855 |
|  | HL | -1.498 | -4.78 – 1.780 | -2.374 | -5.65 – 0.904 | -0.876 | -4.32 – 2.57 | 0.6185 |

**Supplementary Table 3.** Comparison of symmetry indices for peak vertical force (PVF) and vertical impulse (VI) between standing and running treadmill modes in healthy cats (n = 10). Robust linear mixed-effects models were used to evaluate the effect of treadmill mode on symmetry indices. Results are presented as estimated marginal means (Mean) with 95% confidence intervals (CI) for standing and running conditions, estimated mean differences (running - standing), corresponding 95% confidence intervals, and p-values. FL: Forelimb; HL: Hindlimb.
